# Overlooked in Fallon?

**DOI:** 10.1289/ehp.113-a224

**Published:** 2005-04

**Authors:** Christian G. Daughton

**Affiliations:** Henderson, Nevada, E-mail: daughton@gmail.com

The statistical examination of the Fallon childhood cancer cluster by Steinmaus et al. (2004) provides renewed justification for opening a larger window onto the expanse of possibilities regarding a possible cause for this extraordinary cluster. Although the cause(s) might very well be the culmination of simultaneous or sequential exposure to an array of chemical stressors (perhaps in conjunction with nonchemical stressors) at the needed concentrations for sufficient time and during critical windows of vulnerability (as dictated by health and nutritional status, age, sex, genetic susceptibility, etc.), it is worth considering possible new, plausible singular causes until each is ruled out.

Significant resources have been devoted to investigating the childhood cancer cluster discovered in 2000 in Fallon, Nevada (Churchill County). Although the magnitude of this cluster of acute lymphoblastic leukemia (ALL) could be attributed to happenstance, the recent analysis by Steinmaus et al. (2004) shows that such a cluster would be expected to occur in the United States by chance less frequently than every 20,000 years.

A surprising, contemporaneous incidence of ALL also developed in Sierra Vista, Arizona (Cochise County Health Department 2004). Other clusters have occurred in several additional western U.S. rural communities, as well as in various locales worldwide. Of possible significance has been the simultaneous emergence in both Churchill and Cochise counties of an extremely rare form of childhood cancer, rhabdomyosarcoma.

None of the hypothesized causes of the Fallon cluster has withstood scrutiny (with the possible exception of an unknown infectious agent—the “population mixing” hypothesis or “Kinlen theory,” although not supported by the examination of Steinmaus et al. 2004), including exposure to arsenic, tungsten, radiation, and jet fuel. Any hypothesis must account for the important fact that these clusters seem to be limited to a span of several years, after which the incidence subsides. Another commonality seems to be arid agricultural locales that experience periods of drought.

Surprisingly, despite the extensive resources and time devoted to searching for an environmental etiology, no consideration has been devoted to one potential cause that would account for many, if not all, of the aspects of these clusters. Pyrrolizidine alkaloids (PAs) comprise a complex galaxy of highly bioactive natural products. Riddelliine, senkirkine, mono-crotaline, retronecine, heliotridine, jacoline, jacozine, jacobine, seneciphylline, and senecionine are but a few of the numerous PAs produced by a wide spectrum of plants. PA-producing plants (e.g., tansy ragwort, coltsfoot, hound’s tongue), especially *Senecio* species, have long been problematic in the western United States and are well known for livestock poisonings.

Fluctuating levels of PA contamination in the consumer food supply, especially via certain herbal teas (e.g., comfrey), honey (Beales et al. 2004), dairy products, beef, and grains, are a function of drought and harvest or foraging conditions, and therefore exhibit aperiodic cycles of high expression. Churchill County happens to be the center of honey production in Nevada (Churchill Co. 2005; Michigan State University Extension 2002); honey is also produced in Cochise County. Honey has been a particular focus for PA contamination; levels can vary from hive to hive by two or more orders of magnitude within the same foraging location and by time of year. PA-producing plants are particularly prevalent in both counties, where they can contaminate the domestic food supply as weeds; some, such as comfrey, continue to be sold by certain vendors of nutritional supplements and health foods.

Sporadic acute exposures or long-term exposure to low levels (e.g., as little as 10 μg/day) of PAs can lead to delayed toxicity (Australia New Zealand Food Authority 2001; Molyneux et al. 1988) (up to 1 or more years after exposure) and could therefore escape causal suspicion or elude measurement. Levels of metabolites insufficient for overt toxicity in adults could be passed from mothers to fetuses and nursing infants. Maternal transfer would also exempt the liver as the major target for the well-documented toxicity for these chemicals. Furthermore, ALL can originate *in utero* (Jensen et al. 2004). Although best known for their hepatotoxicity (where the bioactive metabolites, such as the dehydropyrrolic products, lead to veno-occlusive diseases and cirrhosis), activated PAs can elicit significant genotoxicity and can be carcinogenic as well as anticarcinogenic (which has led to their experimental use in chemotherapy). Some PA adducts persist in tissues from which metabolites can be released, even long after initial exposure, and migrate to other tissues or can be transported to fetuses or nursing infants (Molyneux and James 1990). It is noteworthy that honey, milk, and grains are also common foods for infants.

Although carcinogenicity data are lacking in humans, PAs have been shown in rats to cause both leukemia (Chan et al. 2003) and rhabdomyosarcoma [California Environmental Protection Agency (EPA) 1999]. The Food and Drug Administration (FDA 2001), the National Toxicology Program (NTP 2003), the World Health Organization (WHO 1988), the California EPA (1999), and others have identified PAs as a major human health threat, especially for fetuses and infants. Significantly, a recent study (Jensen et al. 2004) points for the first time to a link between maternal diet and ALL, where consumption of carotenoids and glutathione (via vegetables) is proposed as being protective. Although linkages of cancer with diet often ascribe the cause to deficiencies or insufficiencies of essential or protective nutrients, just as likely would be the presence of particular chemical stressors—anthropogenic and natural toxicants alike. This finding of Jensen et al. (2004) fits nicely with the fact that glutathione conjugation in particular is known to be a major detoxification route for PAs. A coordinated investigation by epidemiologists, toxicologists, and environmental chemists of a PA–leukemia linkage could prove to be a prudent investment.

## References

[b1-ehp0113-a00224] Australia New Zealand Food Authority 2001. Pyrrolizidine Alkaloids in Food: A Toxicological Review and Risk Assessment. Technical Report Series No. 2. Canberra, Australia:Australia New Zealand Food Authority. Available: http://www.foodstandards.gov.au/_srcfiles/TR2.pdf [accessed 2 March 2005].

[b2-ehp0113-a00224] Beales KA, Betteridge K, Colegate SM, Edgar JA (2004). Solid-phase extraction and LC-MS analysis of pyrrolizidine alkaloids in honeys. J Agric Food Chem.

[b3-ehp0113-a00224] California EPA 1999. Safe Drinking Water and Toxic Enforcement Act of 1986 (Proposition 65). Availability of Draft Data Summaries and Draft Priorities for Chemicals With Respect to Their Potential to Cause Cancer: Request for Relevant Information. Sacramento, CA:California Environmental Protection Agency, Office of Environmental Health Hazard Assessment. Available: http://www.oehha.ca.gov/prop65/pdf/batch3_8.pdf [accessed 1 March 2005].

[b4-ehp0113-a00224] Chan PC, Haseman JK, Prejean JD, Nyska A (2003). Toxicity and carcinogenicity of riddelliine in rats and mice. Toxicol Lett.

[b5-ehp0113-a00224] Churchill Co 2005. Churchill County, Nevada Homepage. Available: http://www.usgennet.org/usa/nv/state/alhn-churchill/alhn-churchill.htm [accessed 2 March 2005].

[b6-ehp0113-a00224] Cochise County Health Department 2004. Leukemia Cluster Information for Cochise County. County of Chochise, Arizona:Cochise County Health Department. Available: http://www.co.cochise.az.us/health/HealthDepartment/Leukemia.htm [accessed 2 March 2005].

[b7-ehp0113-a00224] FDA 2001. FDA Advises Dietary Supplement Manufacturers to Remove Comfrey Products from the Market [letter to industry]. College Park, MD:Center for Food Safety and Applied Nutrition, Food and Drug Administration. Available: http://www.cfsan.fda.gov/~dms/dspltr06.html [accessed 1 March 2005].

[b8-ehp0113-a00224] Jensen CD, Block G, Buffler P, Ma X, Selvin S, Month S (2004). Maternal dietary risk factors in childhood acute lymphoblastic leukemia (United States). Cancer Causes Control.

[b9-ehp0113-a00224] Michigan State University Extension 2002. Agricultural Tourism in Cochise County, Arizona Characteristics and Economic Impacts. Tourism Educational Materials -33839801. Available: http://www.msue.msu.edu/msue/imp/modtd/33839801.html [accessed 2 March 2005].

[b10-ehp0113-a00224] Molyneux RJ, James LF (1990). Pyrrolizidine alkaloids in milk: thresholds of intoxication. Vet Hum Toxicol.

[b11-ehp0113-a00224] Molyneux RJ, Johnson AE, Stuart LD (1988). Delayed manifestation of Senecio-induced pyrrolizidine alkaloidosis in cattle: case reports. Vet Hum Toxicol.

[b12-ehp0113-a00224] NTP 2003. Toxicology and Carcinogenesis Studies of Riddelliine (CAS No.23246-96-0) in F344/N Rats and B6C3F_1_ Mice (Gavage Studies). Technical Report 508. Research Triangle Park, NC:National Toxicology Program. 12844193

[b13-ehp0113-a00224] Steinmaus C, Lu M, Todd RL, Smith AH (2004). Probability estimates for the unique childhood leukemia cluster in Fallon, Nevada, and risks near other U.S. military aviation facilities. Environ Health Perspect.

[b14-ehp0113-a00224] WHO 1988. Pyrrolizidine Alkaloids. Environmental Health Criteria No. 80. Geneva:World Health Organization. Available: http://www.inchem.org/documents/ehc/ehc/ehc080.htm [accessed 1 March 2005].

